# *Helicobacter hepaticus* Infection Promotes the Progression of Liver Preneoplasia in BALB/c Mice *via* the Activation and Accumulation of High-Mobility Group Box-1

**DOI:** 10.3389/fmicb.2021.789752

**Published:** 2022-01-03

**Authors:** Shuyang Cao, Jiancheng Miao, Miao Qian, Chen Zhu, Shiping Ding, Jun Yin, Liqi Zhu, Quan Zhang

**Affiliations:** ^1^College of Veterinary Medicine, Institute of Comparative Medicine, Yangzhou University, Yangzhou, China; ^2^Jiangsu Co-innovation Center for Prevention and Control of Important Animal Infectious Diseases and Zoonoses, Yangzhou University, Yangzhou, China; ^3^Affiliated Hospital of Yangzhou University, Yangzhou University, Yangzhou, China

**Keywords:** *Helicobacter hepaticus*, hepatic preneoplasia, HMGB1, mouse model, mouse (BALB/c)

## Abstract

It has been documented that *Helicobacter hepaticus* (*H. hepaticus*) infection is linked to chronic hepatitis and fibrosis in male BALB/c mice. However, the mechanism underlying the mice model of *H. hepaticus*–induced hepatocellular carcinoma is not fully known. In this study, male BALB/c mice were infected with *H. hepaticus* for 3, 6, 12, and 18 months. *H. hepaticus* colonization, histopathology, expression of proinflammatory cytokines, key signaling pathways, and protein downstream high-mobility group box-1 (HMGB1) in the liver were examined. Our data suggested that the *H. hepaticus* colonization level in the colon and liver progressively increased over the duration of the infection. *H. hepaticus*–induced hepatic inflammation and fibrosis were aggravated during the infection, and hepatic preneoplasia developed in the liver of infected mice at 12 and 18 months post-inoculation (MPI). *H. hepaticus* infection increased the levels of alanine aminotransferase and aspartate aminotransferase in the infected mice. In addition, the mRNA levels of *IL-6*, *Tnf-*α, *Tgf-*β, and HMGB1 were significantly elevated in the liver of *H. hepaticus*–infected mice from 3 to 18 MPI as compared to the controls. In addition, Ki67 was increased throughout the duration of the infection. Furthermore, HMGB1 protein was activated and translocated from the nucleus to the cytoplasm in the hepatocytes and activated the proteins of signal transducers and activators of transcription 3 (Stat3) and mitogen-activated protein kinase (MAPK) [extracellular regulated protein kinases 1/2 (Erk1/2) and mitogen-activated protein kinase p38 (p38)] upon *H. hepaticus* infection. In conclusions, these data demonstrated that male BALB/c mice infected with *H. hepaticus* are prone to suffering hepatitis and developing into hepatic preneoplasia. To verify the effect of HMGB1 in the progression of liver preneoplasia, mice were infected by *H. hepaticus* for 2 months before additional HMGB1 recombinant adenovirus treatment. All mice were sacrificed at 4 MPI, and the sera and liver tissues from all of the mice were collected. Immunology and histopathology evaluation showed that HMGB1 knockdown attenuated the *H. hepaticus*–induced hepatic and fibrosis at 4 MPI. Therefore, we showed that *H. hepaticus*–induced liver preneoplasia is closely correlated with the activation and accumulation of HMGB1.

## Introduction

Liver cancer, which has displayed consistently in incidence and mortality in the past decades, is one of the main causes of cancer death and a major health problem in both developed and developing countries (Torre et al., 2015; Chen W. et al., 2016). The pathogenesis of liver cancer includes complex interactions between the host and the environment. In addition to a disordered lifestyle and behavior habits, infection with pathogenic microorganism, such as hepatitis B virus, hepatitis C virus, and *Schistosoma*, is also a potential factor known to enhance the risk of cancer (Ringelhan et al., 2017). It has been reported that these pathogens are the causes of many chronic liver diseases including hepatocellular carcinoma (HCC), which is the second leading cause of death from malignancy following lung cancer (Ferlay et al., 2015). In addition, because of the close anatomical and physiological connection between the gut and liver, gut microbiota are thought to be one of the triggers of chronic liver diseases and HCC (Milosevic et al., 2019). Several studies have revealed that *Helicobacter* species, a class of intestinal pathogenic bacteria, were found in the tissue and serum samples among cirrhotic subjects and patients with hepatic carcinoma (Spinzi et al., 2001; Fox et al., 2010), suggesting that animals infected with *Helicobacter* species may prove to be a new model to investigate the mechanisms of gut microbiota–associated liver carcinogenesis.

*Helicobacter hepaticus*, a member of the *Helicobacter* species, might contribute to the progression of various liver diseases in humans (Hamada et al., 2009). Serodiagnosis and immunoblotting assays have implicated *Helicobacter hepaticus* (*H. hepaticus*) infection in the pathogenesis of hepatitis, liver cirrhosis, and HCC (Murakami et al., 2011). However, *H. hepaticus* has yet to be isolated from any liver samples of patients suffering from hepatic diseases (Yang et al., 2013), suggesting that the condition of artificial culture *in vitro* is not mature enough. *H. hepaticus* infection has been recognized to result in chronic active hepatitis similar to that seen in humans, which may lead to the formation and development of hepatobiliary carcinogenesis in certain strains of mice (A/JCr, 129/SvEv, Rag2^–/–^, and BALB/c mice) (Fox et al., 1996). Furthermore, it has been documented that various rodent strains have shown a link between *H. hepaticus* infection and host immunity in disease pathogenesis (Whary et al., 2001; Garcia et al., 2008), which manifests in a manner similar to patients with hepatic diseases.

Our previous study demonstrated that *H. hepaticus* infection induced severe liver inflammation and fibrosis in male BALB/c mice at 6 months post-inoculation (MPI), which was dependent upon hepatocytes releasing and transferring a cytokine, interleukin-33 (IL-33), to the cytoplasm and extracellular region (Cao et al., 2020a,b). In addition, released IL-33 plays a pivotal role as the first pro-inflammatory cytokine to sound the alarm during hepatocellular necrosis or damage to the barrier of the liver (Moussion et al., 2008). High-mobility group box-1 (HMGB1), another alarmin, shares many characteristics with IL-33, including cellular localization, functions, and involvement in various hepatic pathologies, such as HCC (Arshad et al., 2012; Suren et al., 2014). We hypothesized that excessive extracellular HMGB1 would aggravate chronic hepatic injury during the development of *H. hepaticus*–induced liver fibrosis in BALB/c mice. Here, we demonstrated, for the first time, that *H. hepaticus* infection induced liver preneoplasia in male BALB/c mice and found that HMGB1 was robustly activated in the liver during *H. hepaticus* infection. In summary, a murine *H. hepaticus* infection model in mice on a BALB/c background has the potential to be used in research to simulate the development of human hepatic carcinogenesis, which would allow for the exploration into mechanisms underlying the pathogenesis of hepatic preneoplasia.

## Materials and Methods

### Mice and Bacterial Strains

Forty male BALB/c mice (free of *Helicobacter* spp. parasites and exogenous murine viral pathogens) were bred and maintained in a specific pathogen-free facility. The animal experiments were conducted in line with China Laboratory Regulation Act (2017) under a Project License [SYXK(SU)2017-0044] authorized by Jiangsu Provincial Science and Technology Department and approved by Institutional Animal Care and Use Committee of Yangzhou University. Mice with free access to food and water were maintained in a room at 22–26°C with 40–60% relative humidity under a 12-h:12-h light-to-dark cycle. *H. hepaticus* 3B1 (ATCC 51449) was cultured on Brucella agar plates (BD, United States) supplemented with 5% sheep blood and antibiotics (amphotericin B, vancomycin, and cefoperazone) for 4–5 days under microaerobic conditions (85% N_2_, 10% CO_2_, and 5% O_2_) at 37°C.

### Experimental Design One

*Helicobacter hepaticus* 3B1 (ATCC 51449) was collected from plates and then resuspended in sterile PBS. The optical density (OD) of the culture was measured using a spectrophotometric, and the concentration of the bacteria for oral gavage was adjusted to 1 × 10^9^ colony-forming unit (CFU)/ml on the basis of the OD_600_ as described previously (Xu et al., 2018). Forty 6-week-old male BALB/c mice were randomly divided into two groups (*n* = 20). One group was orally gavaged with *H. hepaticus* 3B1, another group was sham-dosed with PBS as a control. Mice were inoculated with 0.2 ml of fresh bacterial suspension by gastric gavage every other day for three times. Five mice from the *H. hepaticus*–infected and control groups were euthanized at 3, 6, 12, and 18 MPI. Serum and liver tissue from all of the mice were collected and processed as previously described (Cao et al., 2020a).

### Biochemical Analysis

Blood from all of the mice was separated by centrifugation at 2,000 × *g* for 15 min at 4°C, and then, the serum was collected. Serum alanine aminotransferase (ALT), aspartate aminotransferase (AST), and hyaluronic acid (HA) levels were determined using a Nergy H1 Microplate Reader (BioTek, United States) and the corresponding kit (Nanjing Jiancheng Biology Engineering Research Institute Nanjing, China).

### Histopathology

Colon and liver tissues were dissected, embedded in paraffin, cut into 5-μm-thick sections, and stained with hematoxylin and eosin (H&E) for morphological and evaluation. Sections of liver tissues were also stained with Sirius Red to assess the area and degree of hepatic fibrosis. The percentage of collagen area in the tissue sections was determined using ImageJ software. Tissue sections were scored by a boarded veterinary pathologist who was blinded to sample identity. Liver sections representing replicate samples from all mice was graded on a scale of 0–4 for hepatitis including lobular and portal hepatitis and staged on the same scale for fibrosis using criteria established by Scheuer (Scheuer et al., 2002; Rogers et al., 2004). A colon histology activity index (HAI) was generated by combining scores for all sub-categorical including inflammation, edema, epithelial defects, crypt atrophy, hyperplasia, and dysplasia, which were graded on a scale of 0–4 as previously described (Erdman et al., 2009).

### Real-Time Quantitative PCR for *Helicobacter hepaticus*

Quantitative was performed to quantify colonization levels of colonic and hepatic *H. hepaticus* 3B1. The abundance of *H hepaticus* in the liver and colon was determined by using CdtB gene primers (F: atgaaagagactttattgcttca, R: agcctgtgcataccctcata) and the SYBR Green Master Kit (Roche, Mannheim, Germany) in an Applied Biosystems StepOne Real-Time PCR System. Genome copy numbers of the *H. hepaticus* were expressed per microgram of murine chromosomal DNA as previous described (Ge et al., 2005).

### Quantitative PCR Analyses of Hepatic Cytokines

Total RNA from the caudate lobe of each liver tissue at 3–18 MPI was extracted, respectively, using TRIzol (Invitrogen, Carlsbad, CA, United States) following the recommendation of the manufacturer. Briefly, 100 mg of frozen liver fragments were homogenized in 1 ml of TRIzol by grinding the tissue into a fine powder with liquid nitrogen in a prechilled mortar. Total RNA was collected using water treated with diethyl pyrocarbonate (DEPC) (DNase free, RNase free, and Proteinase free). cDNA was synthesized from 1 μg of total RNA using a PrimeScript RT reagent with the gDNA eraser kit (Takara, Dalian, China). Transcript levels of hepatic *IL-6*, *Tgf-*β, *Tnf-*α, and HMGB1 genes were amplified by qPCR using the Faststart Universal SYBR Green Master (ROX) (Roche, Mannheim, Germany), and the mRNA level of the β-actin gene in each cDNA sample was measured and used for normalization. qPCR primers (*IL-6*: F: gagaggagacttcacagagg, R: gtactccagaagaccagagg; *Tnf-*α: F: catcttctcaaaattcgagtgacaa, R: tgggagtagacaaggtacaaccc; *Tgf-*β: F: tgcgcttgcagagattaaaa, R: ctgccgtacaactccagtga; HMGB1: F: ggactctcctttaaccgc, R: ttgtgatagccttcgctggg; and β-actin: F: aaagacctgtacgccaacac, R: gtcatactcctgcttgctgat) were synthesized by Shanghai Shenggong Biotech Co., Ltd. All samples were run in triplicate and the concentration of cDNA was 100 ng/μl. The qPCR cycling program was at 95°C for 30 s, followed by 40 cycles of 94°C for 5 s and 60°C for 30 s. The mean values of the triplicates from each sample and the relative expression level of each sample were calculated. Relative expression levels were calculated using the 2^−ΔΔCt^ method.

### Western Blot Analysis

Total proteins were isolated from the left lobe of each liver tissue. Proteins of cytoplasm were obtained using a Cytoplasmic Protein Preparation Kit (APPLYGEN, Beijing, China). Twenty micrograms of proteins of each sample was loaded onto a 10% Sodium dodecyl sulfate-polyacrylamide gel electrophoresis (SDS-PAGE), separated by electrophoresis, and transferred to poly vinylidene fluoride (PVDF) membranes. The membranes were incubated with the respective primary antibodies, including Stat3, phospho-Stat3, Erk1/2, phospho-Erk1/2, p38, phosphor-p38, α-SMA (1:1,000, Cell Signaling Technology, United States), and HMGB1 (1:1,000, Abcam, United States) for 16 h at 4°C. Subsequently, the membranes were incubated with secondary antibodies conjugated to horseradish peroxidase for 1 h. The proteins were visualized by Amersham ECL Select Western Blotting Detection Reagent.

### Immunohistochemistry

Mice liver tissues embedded in paraffin were cut into 5-μm sections. The liver sections were required an antigen retrieval with citrate and incubated with primary antibodies of alpha smooth muscle actin (a-SMA) (1:200, Wuhan Servicebio Technology Co., Ltd., China), Ki67 (1:200, Abcam, United States), alpha-fetoprotein (AFP) (1:1,000, Abcam, United States), collagen I (1:200, Wuhan Servicebio Technology Co., Ltd., China), and HMGB1 (1:400, Abcam, United States) for 16 h at 4°C. The sections were developed with 3,3N-diaminobenzidine tertrahydrochloride (DAB) (VECTOR, United States). The nuclei were counterstained with hematoxylin for 1 min. These sections were observed under a light microscope.

### ELISA Detection

Released HMGB1 in serum was detected using a specific ELISA kit (Solarbio, Beijing, China) with a microplate reader according to the instructions of the manufacturer.

### Experimental Design Two

About 6-week-old male BALB/c mice were randomly divided into two groups, namely, the *H. hepaticus* infection group (*H. h*) and the group both infected by *H. hepaticus* and injected with AdshHMGB1 (AdshHMGB1 + *H. h*). Each group included five mice. Detailed experiments were as described before (Cao et al., 2020b). After all mice were sacrificed at 4 MPI, the sera and liver tissues from all of the mice were collected. Sections of liver tissues were also stained with Sirius Red.

### Statistical Analysis

All statistical analyses were performed using SPSS statistical software. Generally, the colonization levels of *H. hepaticus* in tissues, cytokine mRNA levels, protein expression levels, and serum biochemistry were analyzed using two-tailed Student’s *t*-tests, and differences were considered statistically significant when *p* < 0.05.

## Results

### *Helicobacter hepaticus* Infection Promoted Colonic and Hepatic Inflammation in BALB/c Mice

**FIGURE 1:**
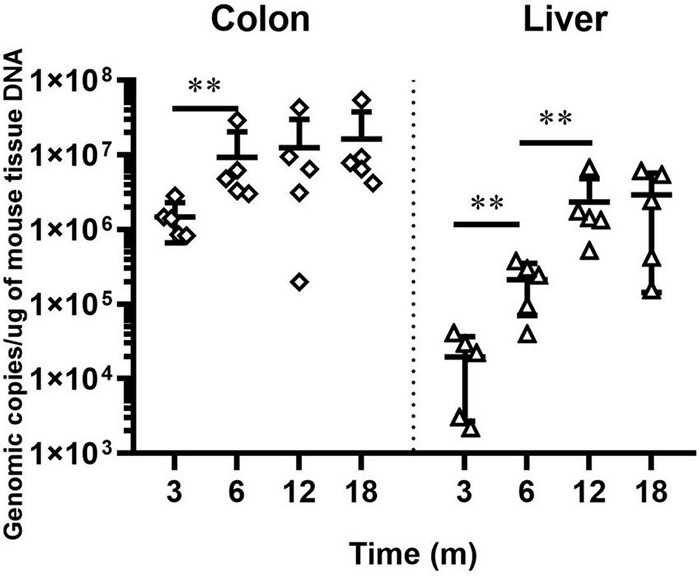
QPCR-based quantitation of the *Helicobacter hepaticus cdtB* gene in intestinal and hepatic segments. Copy numbers of the *H. hepaticus* genome are expressed per micrograms of mouse DNA in the respective samples. The samples of control groups negative for *H. hepaticus* are not shown. ^**^p < 0.01 as compared to the control. Each group contained five mice.

We previously documented that *H. hepaticus* colonization level increased from 8 to 24 weeks post-infection (WPI). We also showed that *H. hepaticus* infection aggravated pathological lesions in the intestine and liver of *H. hepaticus*–infected BALB/c mice (Cao et al., 2020a). To better elucidate the relationship between bacterial load and disease development, we determined the level of *H. hepaticus* colonization in the colon and liver of infected BALB/c mice until 18 MPI. qPCR results revealed that *H. hepaticus* was detected in the colon and liver of *H. hepaticus*–infected BALB/c mice at all the time points. However, there was no statistical difference in colonization level in the colon between 6 and 18 MPI and in the liver between 12 and 18 MPI (Figure 1).

**FIGURE 2:**
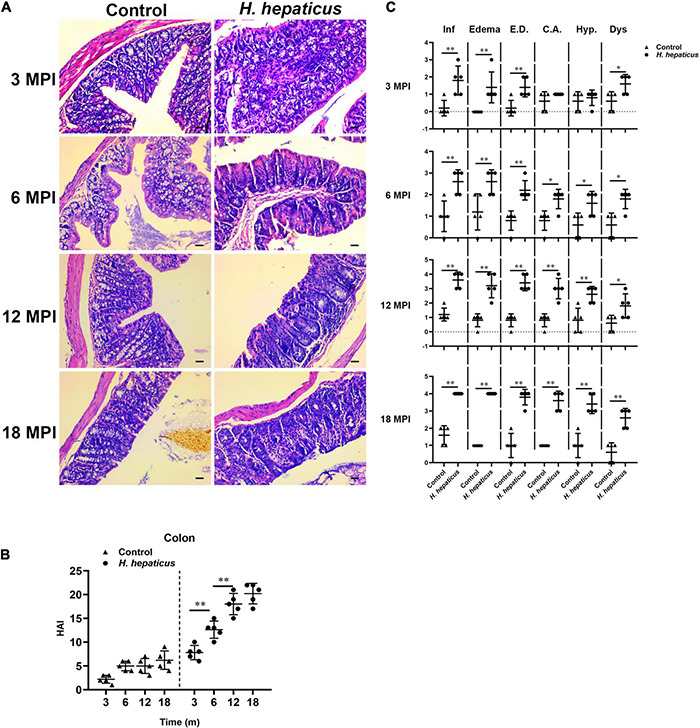
*Helicobacter hepaticus* infection induced chronic colitis. **(A)** H&E images of the colon from male mice of control and *H. hepaticus*–infected groups at 3, 6, 12, and 18 months (magnification, × 200). **(B)** The intestinal histologic active index (HAI) was calculated by combining scores for all sub-categorical lesions of mice in the control and *H. hepaticus*–infected groups at all time points. **(C)** Scores of sub-categorical lesions in the colons in all representative mice at all time points. Data are expressed as the means ± SD (*n* = 5). *p < 0.05 as compared to the control, and ^**^p < 0.01 as compared to the control. Each group contained five mice.

Consistent with previous reports (Cao et al., 2020a), mice infected with *H. hepaticus* developed overt colonic pathology including disruption of the epithelial layer along with lymphocytes infiltration, as well as the loss of colonic glands, which extended throughout the entire colon during the later stages of infection (Figure 2A). The colonic pathology HAI score was significantly higher in the *H. hepaticus*–infected mice as compared to the control mice at all infection points (Figure 2B). Meanwhile, the *H. hepaticus*–infected mice at 12 MPI had statistically higher colonic HAI scores as compared to that at 3 and 6 MPI. However, the colonic HAI scores at 18 MPI were comparable to that at 12 MPI. For sub-categorical colonic lesions including inflammation, edema, crypt atrophy, epithelial defects, hyperplasia, and dysplasia, *H. hepaticus* infection groups developed more severe inflammation, edema, and epithelial defects at all time points as compared with control groups (Figure 2C). These results indicated that infection with *H. hepaticus* promoted chronic colonic inflammation.

**FIGURE 3:**
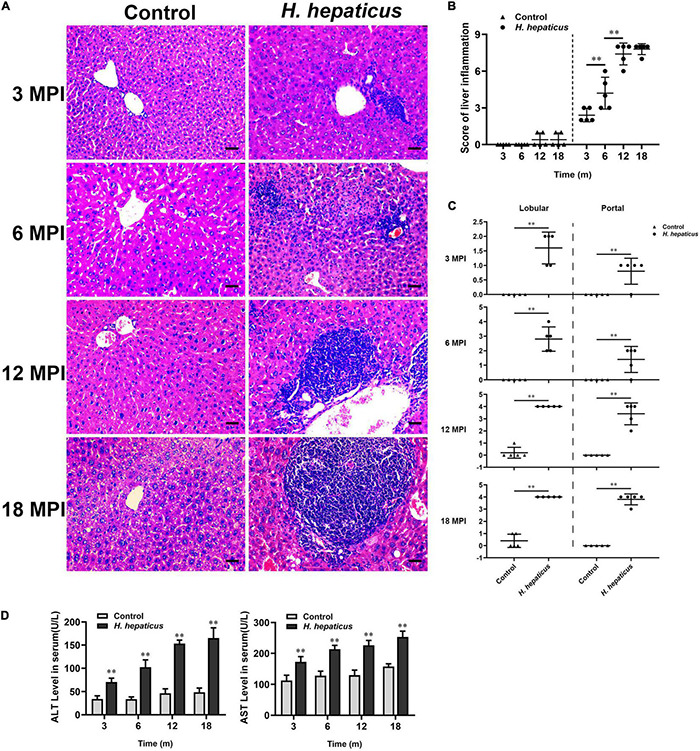
*Helicobacter hepaticus* infection induced chronic hepatitis. **(A)** H&E images of the liver from male mice of control and *H. hepaticus*–infected groups at 3, 6, 12, and 18 months (magnification, × 200). **(B)** The scores of the degree of hepatic inflammation were calculated by combining the sub-categorical hepatitis scores of mice in the control and *H. hepaticus*–infected groups at all time points. **(C)** Scores of sub-categorical lesions of liver in representative mice at all time points. **(D)** The levels of serum ALT and AST activity between the *H. hepaticus*–infected groups and control groups. Data are expressed as the means ± SD (*n* = 5). ^**^p < 0.01 as compared to the control. Each group contained five mice.

To determine potential correction of hepatic lesions with *H. hepaticus* colonization in the liver of BALB/c mice, we next examined hepatic histopathology. Inflammatory lesions in the liver involved two aspects: lobular (acinar) hepatitis and/or portal hepatitis. These pathological manifestations consisted mainly of lymphocytes forming foci of spotty or confluent hepatocellular necrosis with Kupffer cells and neutrophil granulocytes (Figure 3A), which was similar to the features of human chronic active and chronic persistent hepatitis (Brunt, 2000). Consistent with *H. hepaticus* infection in A/JCr mice, lobular hepatitis lesions were evident at all sampled time points, whereas chronic portal inflammation was not fully prominent until 12 MPI (Rogers et al., 2004). To quantify the extent of hepatic lesions, the grading scores of separate components (lobular and portal activity) were added (Figure 3B). The results indicated that the *H. hepaticus*–infected mice shared significantly higher hepatic inflammatory scores as compared to the control mice at all time points. Moreover, the severity of *H. hepaticus*–induced hepatitis increased over the course of infection although the scores of hepatitis exhibited no statistical difference between 12 and 18 MPI. For sub-categorical hepatic lesions including lobular hepatitis and portal hepatitis, lobular hepatitis occurred before portal hepatitis (Figure 3C). Next, biochemical criteria, such as ALT and AST levels, were used as markers to determine liver function (Figure 3D). Increased serum ALT and AST levels were evident in *H. hepaticus*–infected mice as compared to the control mice, suggesting that mice infected with *H. hepaticus* endured liver damage.

### Infection With *Helicobacter hepaticus* Aggravated Hepatic Fibrosis in BALB/c Mice

**FIGURE 4:**
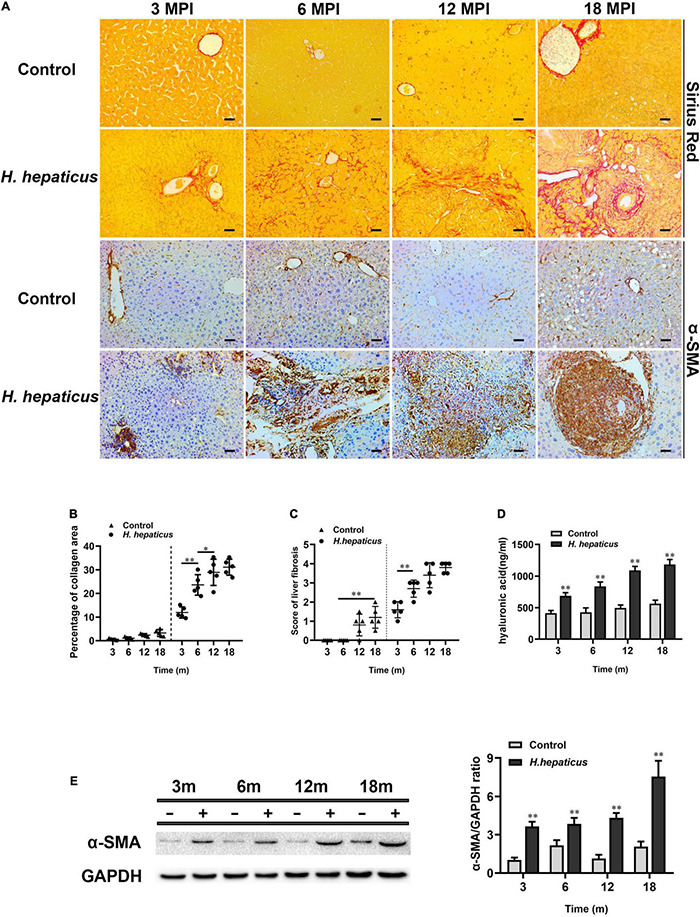
*Helicobacter hepaticus* infection induced liver fibrosis. **(A)** Sirius red staining and immumohistochemical staining (IHC) of α-SMA images of liver sections at all time points (magnification, × 200). **(B)** Quantitative morphometry of collagen positive area. **(C)** The scores of the degree of liver fibrosis. **(D)** The levels of serum hyaluronic acid activity between the *H. hepaticus*–infected groups and control groups. **(E)** The expression level of α-SMA was detected and analyzed by Western blot. Data are expressed as the means ± SD (*n* = 5). *p < 0.05 as compared to the control. ^**^p < 0.01 as compared to the control. Each group contained five mice.

The expression of α-SMA is usually deemed as a sign of activated hepatic stellate cells (HSCs), which is the key event in the process of liver fibrosis (Dhar et al., 2020). The immunohistochemistry of α-SMA results indicated that more severe hepatic fibrosis was observed in *H. hepaticus*–infected mice livers as compared to the control mice (Figure 4A). Sirius Red staining results showed the distensible collagen area in BALB/c mice over the course of infection (Figure 4B). The liver fibrosis stage was 1.6 ± 0.418 at 3 MPI and progressed to 3.8 ± 0.274 at 18 MPI in mice infected with *H. hepaticus* (Figure 4C). However, because of the differences in staining sensitivity, Sirius Red staining embodied little difference in liver fibrosis between 12 and 18 MPI. Next, the increased level of serum hyaluronic acid was higher in *H. hepaticus*–infected mice as compared with the control mice (Figure 4D). Moreover, our data also showed that the expression of α-SMA was significantly elevated in the liver of *H. hepaticus*–infected mice compared to the control group (Figure 4E). Remarkably, uninfected BALB/c mice also showed mild liver fibrosis at 12 and 18 months, suggesting aged mice were at risk to spontaneously form liver fibrosis.

### *Helicobacter hepaticus* Infection Promoted Hepatocytes and Immunocytes Proliferation

**FIGURE 5:**
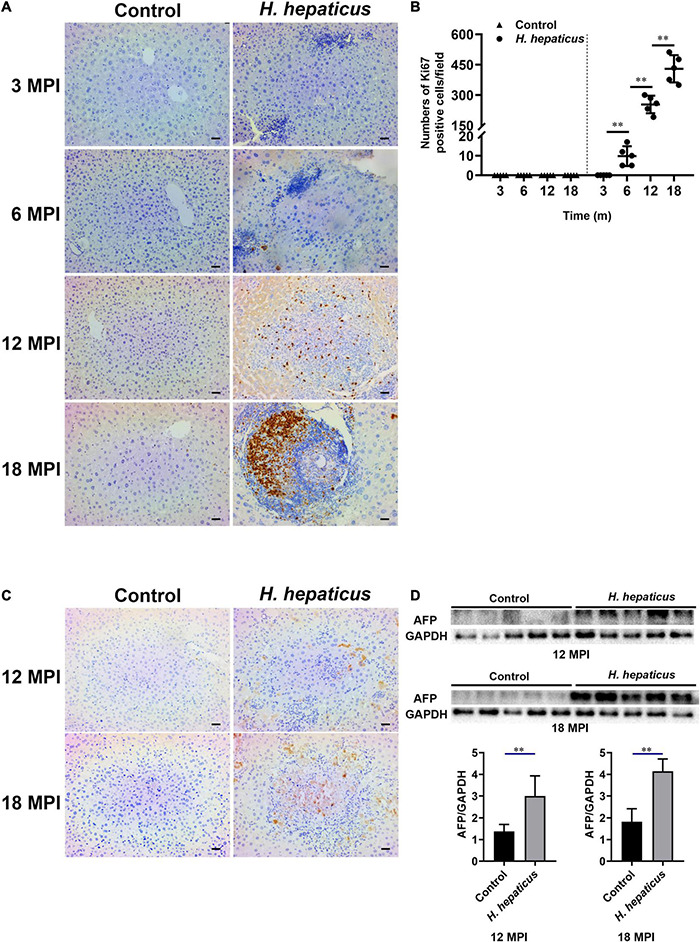
*Helicobacter hepaticus* infection induced cell proliferation and hepatic preneoplasia. **(A)** Immumohistochemical (IHC) staining of Ki67 images of the liver in *H. hepaticus*–infected and control mice at all time points (magnification, × 200). **(B)** The number of Ki67-positive cells was calculated and compared. Data are expressed as the means ± SD (*n* = 5). **(C)** Immumohistochemical (IHC) staining of AFP images of the liver in *H. hepaticus*–infected and control mice at 12 and 18 MPI (magnification, × 200). **(D)** Western blot and densitometric analysis of AFP and GAPDH of the liver in *H. hepaticus*–infected and control mice at 12 and 18 MPI. ^**^p < 0.01 as compared to the control. Each group contained five mice.

Ki67 expression level indicates the status of cell proliferation, which is highly over expressed in cancer cells and has been proposed as a diagnostic marker of cancer (Yang et al., 2018). Immunostaining for Ki67 expression is the gold standard. A cutoff level of 10–14% positive staining is used to judge high risk of prognosis (Yang et al., 2018). Immunohistochemistry results showed that Ki67 was first visible in the liver of mice infected with *H. hepaticus* at 6 MPI and also evident in foci of cellular alterations and dysplastic nodules at 12 and 18 MPI (Figure 5A). The Ki67 labeling index per × 10 field was calculated in tertiary lymphoid tissue at different time points (Figure 5B), and the number of proliferating cells in *H. hepaticus*–infected groups was significantly increased compared with the control. AFP possesses a variety of biological functions, including its diagnosis in liver cancer (Wang and Wang, 2018). The immunohistochemistry results showed that AFP was visible in the liver of mice infected with *H. hepaticus* at 12 and 18 MPI (Figure 5C). Furthermore, immunoblot results showed that the level of AFP in the liver of mice in *H. hepaticus*–infected groups was higher as compared with controls (Figure 5D). Together, these lines of evidence demonstrated that *H. hepaticus* infection aggravated hepatic damage, which developed into hepatic carcinoma genesis.

### *Helicobacter hepaticus* Infection Increased the Expression of Hepatic Inflammatory Cytokines

**FIGURE 6:**
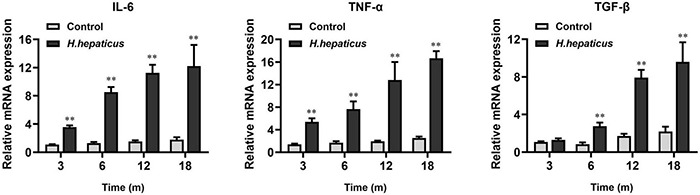
*Helicobacter hepaticus* infection upregulated the levels of inflammation-associated cytokines. Relative mRNA levels of hepatic *IL-6*, *Tnf-*α, and *Tgf-*β. Data are expressed as the means ± SD (*n* = 5). ^**^p < 0.01 as compared to the control. Each group contained five mice.

We previously reported that *H. hepaticus* infection caused liver inflammation and a significant upregulation of hepatic *IL-6* and *Tnf-*α levels in male BALB/c mice starting at 8 WPI (Cao et al., 2020a). Likewise, the mRNA levels of these inflammatory cytokines were significantly increased as compared to the controls at 12 and 18 MPI (Figure 6) in the current study. In addition, it has been documented that *Tgf-*β family members (*Tgf-*β*-1*, *Tgf-*β-β*-2*, and *Tgf-*β-β*-3*) are induced and activated in a variety of fibrotic diseases (Govinden and Bhoola, 2003). In this study, we found that the mRNA levels of the fibrosis-related hepatic cytokine *Tgf-*β were statistically different between the *H. hepaticus*–infected and control groups at all time points.

### *Helicobacter hepaticus* Infection Induced Hepatic Preneoplasia *via* the Activation and Release of High-Mobility Group Box-1, MAPKs (Erk1/2 and p38), and Stat3

**FIGURE 7:**
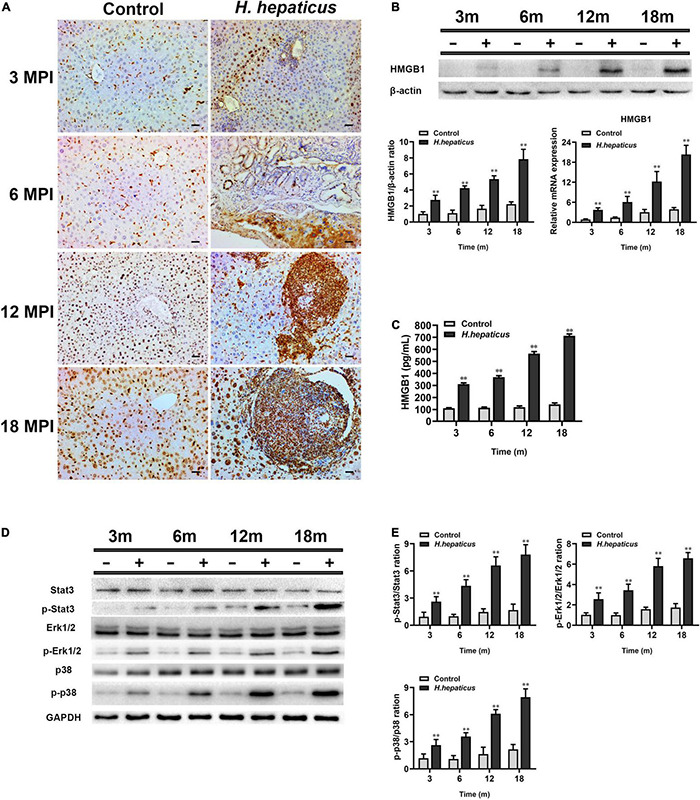
*Helicobacter hepaticus* infection promoted activation and release of HMGB1. **(A)** Immumohistochemical (IHC) staining of HMGB1 images in the liver of *H. hepaticus*–infected and control mice at all time points (magnification, × 200). **(B)** The mRNA and protein expression level of HMGB1 was detected and analyzed. **(C)** The level of serum HMGB1 was detected and analyzed. **(D,E)** Western blot and densitometric analysis of Stat3, p-Stat3, Erk1/2, p-Erk1/2, p38, p-p38, and β-actin. Data are expressed as the means ± SD (*n* = 5). ^**^p < 0.01 as compared to the control. Each group contained five mice.

It has been documented that HMGB1 plays a key role in biological processes that contribute to characters of chronic liver diseases and may serve as a marker for monitoring the surgical course in patients undergoing surgery for liver cancer (Hernandez et al., 2018; Cheng et al., 2020; Satoh et al., 2020). To investigate whether *H. hepaticus*–induced hepatic preneoplasia was associated with HMGB1, the level of HMGB1 in infected mice was detected. Immunohistochemical results demonstrated that *H. hepaticus* infection facilitated HMGB1 localization in the hepatocytes and immune cells at each time point (Figure 7A). Of note, the livers of control mice showed an increased HMGB1 signal in the nucleus of parenchymal cells with age, but hepatic architecture was normal. In addition, the mRNA and protein levels of HMGB1 were examined in the liver of both *H. hepaticus*–infected mice and controls at 3, 6, 12, and 18 MPI utilizing qPCR and Western blotting. Our results showed that both the mRNA and protein levels of HMGB1 were statistically increased in the *H. hepaticus*–infected mice as compared to the controls at all time points (Figure 7B). Furthermore, HMGB1 in serum was significantly increased further suggesting that hepatocytes were damaged upon *H. hepaticus* infection (Figure 7C). Because MAPK (Erk1/2 and p38) and Stat3 signaling pathways have been characterized in downstream of HMGB1 (Zhang et al., 2021), we investigated the mechanisms of *H. hepaticus*–induced hepatic preneoplasia and HMGB1 stimulation. The results indicated that phosphorylation of hepatic Erk1/2, p38, and Stat3 were significantly increased from 3 to 18 MPI in *H. hepaticus*–infected mice as compared to the controls (Figures 7D,E). Our data suggested that the activation of these proteins was correlated with progression of *H. hepaticus*–induced hepatic pathology in this mouse model.

### Knockdown of High-Mobility Group Box-1 Could Alleviate Liver Fibrosis in BALB/c Mice Infected With *Helicobacter hepaticus*

**FIGURE 8:**
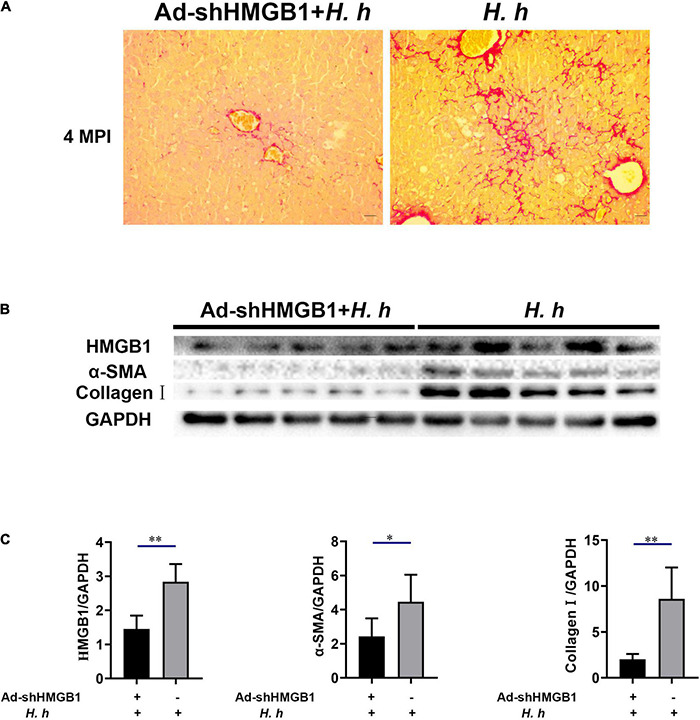
Knockdown of HMGB1 expression could alleviate liver fibrosis in BALB/c mice infected with *H. hepaticus* at 4 MPI. **(A)** Sirius red staining of liver sections including AdshHMGB1 + *H. h* group and *H. h* group at 4 MPI. **(B,C)** Western blot and densitometric analysis of hepatic HMGB1, α-SMA, collagen I, and GAPDH of AdshHMGB1 + *H. h* group and *H. h* group at 4 MPI. Data are expressed as the means ± SD (*n* = 5). *p < 0.05 and ^**^p < 0.01 as compared to the control. Each group contained five mice.

To prove the effect of HMGB1 in liver fibrosis, we knocked down the expression of HMGB1 in the liver of mice by adenovirus interference. The AdshHMGB1 + *H. h* group showed a mild liver fibrosis compared with the *H. h* group at 4 MPI (Figure 8A). Collagens are the principal components of the fibrotic scars that develop as a result of liver fibrosis (Karsdal et al., 2020). The immunoblot results showed knockdown of HMGB1 could decrease the expression of hepatic α-SMA and collagen I in BALB/c mice infected with *H. hepaticus* at 4 MPI (Figures 8B,C), suggesting the release of HMGB1 influenced the progression of liver fibrosis.

## Discussion

The liver performs an integral role in the body to maintain homeostasis and health and is a major synthesis and immunological organ with hundreds of functions such as metabolism, antigen surveillance, and immune tolerance (Tanwar et al., 2020). It has been documented that the liver is at high risk of suffering stimulation, injury, inflammation, and developing cancer by enhancing proliferation ability of the damaged cells (Finkin et al., 2015; Zhan et al., 2016). *H. hepaticus* has been shown to cause HCC in susceptible mouse strains (Fox et al., 1996), but the underlying mechanisms were obscure. We previously reported that *H. hepaticus* infection induced liver inflammation and cirrhosis in BALB/c mice by 6 MPI (Cao et al., 2020a). In this study, our data further demonstrated that *H. hepaticus* infection induced chronic hepatitis/cirrhosis progressed to hepatic preneoplasia by 18 MPI in this mouse model, whereas no obvious lesions was found in the livers of uninfected aged mice. Likewise, the worldwide morbidity and mortality of liver cancer in humans are influenced by age, environment, and immunity (Berruti et al., 2019; Franken et al., 2019). Previous studies have shown that *H. hepaticus* infection was associated with colitis and colorectal cancer in some immunodeficient mice. In this study, increased *H. hepaticus* in the colon only induced chronic colitis without cancer and tumor in male BALB/c mice during 18 months of infection, indicating that adaptive immune cells of immunocompetent mice normally serve to reinforce homeostasis and prevent cancer in the lower bowel (Fox et al., 2011). However, *H. hepaticus* persisted as a lifelong infection in the colon of BALB/c mice, which probably induced damage of the intestine epithelial barrier and transfer to the liver (Segura-Lopez et al., 2015). Infiltration of immune cells usually appeared in the livers of *H. hepaticus*–infected mice that developed liver cancer, indicating that chronic inflammation might progress to carcinogenesis(Han et al., 2019). In the current study, *H. hepaticus* infection in male BALB/c mice induced chronic hepatic inflammation throughout the process by stimulating the secretion of pro-inflammatory cytokines such as *IL-6*, *Tnf-*α, and *Tgf-*β, which resulted in the development of hepatic preneoplasia. Moreover, the released cytokines could activate HSCs, the main mediator of liver fibrosis (Seki et al., 2007). In addition, the degree of liver fibrosis in *H. hepaticus*–infected mice as revealed by Sirius Red staining and α-SMA analysis was more serious as compared to the controls and reached a high level at 12 MPI. In contrast to a previous study where male A/JCr mice infected with *H. hepaticus* presented with features of liver carcinoma at 12 MPI, no hepatic fibrosis was observed during the middle course of infection (Rogers et al., 2004), suggesting that different mouse strain may have different pathological characteristics. It is noteworthy to mention that the aged control mice were still at risk of developing mild liver fibrosis with age. Together, these lines of evidence are consistent with previous points that *H. hepaticus* infection in male BALB/c mice recapitulated some pathological features in the development of HCC in humans, which indicates that this model is suitable for anatomizing the mechanisms on human HCC caused by gut bacterial infection.

Ki67, a nuclear antigen expressed only in proliferating cells, is one of the most widely used proliferation markers in cancer cells linked with HCC (Shi et al., 2015; Sun and Kaufman, 2018). Our investigations of samples *in vivo* revealed that the number of Ki67 positive cells was much higher in the liver of *H. hepaticus*–infected mice than that of the controls after 12 MPI. These results are in agreement with a previous study that showed increased cell proliferation was also evident in liver of A/JCr mice with *H. hepaticus* infection (Rogers et al., 2004), indicating that *H. hepaticus* infection promoted proliferation of hepatic cells and probably induced hepatic tumorigenesis in male BALB/c mice. It has been reported that cytolethal distending toxin (CDT) plays a key role in arresting cell cycles and induces inflammation and hepatic carcinogenesis in *H. hepaticus*–infected mice (Ge et al., 2007), which may be a reason for the increase and accumulation of Ki67 in hepatocytes. In brief, our findings suggested that excessive cellular proliferation occurred in *H. hepaticus*–induced hepatic preneoplasia, which is feasible because longer infection duration is essential for the development of HCC in this model. High-mobility group box-1 is a typical damage-associated molecular pattern molecule and has been implicated in several inflammatory and cancerous diseases (Sims et al., 2010). It has been reported that the serum HMGB1 levels in patients with HCC were significantly higher than those with single chronic hepatitis and healthy controls (Tang et al., 2010), indicating that HMGB1 can be an important and reliable serous marker of chronic liver diseases and used to predict the processes in HCC patients. In this study, we observed that accumulation of HMGB1 was increased and passively released in the liver from necrotic hepatocytes, which, in turn, could activate monocytes, macrophages, and dendritic cells in the area of inflammation, and then promoted an intratumoral microenvironment favoring cell growth and proliferation (Naglova and Bucova, 2012). It has been reported that HMGB1 can be released to extracellular context by necrotic cells under hypoxia in growing solid tumor (Wang and Zhang, 2020). Then, extracellular HMGB1 promotes the release of cytokines such as *IL-6* and *Tnf-*α by activating MAPKs and Stat3 pathways, which stimulates tumor cells proliferation, angiogenesis, EMT, invasion, and metastasis (Chen Y. et al., 2016; Bailly, 2020). Our data also showed that *H. hepaticus* infection upregulated these cytokines and the phosphorylation of Erk1/2, p38, and Stat3 in the livers of *H. hepaticus*–infected BALB/c mice at all time points, indicating that *H. hepaticus* infection induced these signaling pathways, and, in turn contributed to promoting liver carcinogenesis (Deng et al., 2019). Moreover, immumohistochemical staining results showed that *H. hepaticus* infection caused HMGB1 activation and translocation from the nucleus to the cytoplasm to promote liver carcinogenesis. In this study, we also demonstrated that knockdown of HMGB1 expression could alleviate liver fibrosis in BALB/c mice infected with *H. hepaticus* at 3 and 4 MPI. However, it was still unknown whether decreased HMGB1 ameliorated the development of liver carcinoma in the murine *H. hepaticus* infection model. Of note, the HMGB1 inside nucleus has intrinsic property to improve its different biological function, such as transcription, regulation of chromatin structure, and DNA damage repair (Tripathi et al., 2019). In this study, although the gene and protein levels of HMGB1 in the hepatocellular nucleus of control mice were increased, no obvious liver damage was found, implying that HMGB1 in the nucleus had no ability to cause a cascade of reactions. Together, *H. hepaticus* infection promotes hepatitis to develop hepatic preneoplasia by activation and accumulation of HMGB1.

In summary, our findings demonstrated that *H. hepaticus* infection in male BALB/c mice induces chronic hepatitis which progresses to liver preneoplasia. This pathogenic process is likely modulated *via* accumulation and release of HMGB1, which offers mechanistic clues for future studies.

## Data Availability Statement

The original contributions presented in the study are included in the article/Supplementary Material, further inquiries can be directed to the corresponding authors.

## Ethics Statement

The animal study was reviewed and approved by the Institutional Animal Care and Use Committee (IACUC) of Yangzhou University.

## Author Contributions

SC processed the data and wrote the manuscript. JM and MQ contributed to the conception of the study. CZ conducted some animal experiments. SD conducted the construction of Adenovirus. JY performed the pathological score. LZ carried out the histological and immunoblotting experiments. QZ checked and modified the manuscript. All authors contributed to manuscript revision, read, and approved the submitted version.

## Conflict of Interest

The authors declare that the research was conducted in the absence of any commercial or financial relationships that could be construed as a potential conflict of interest.

## Publisher’s Note

All claims expressed in this article are solely those of the authors and do not necessarily represent those of their affiliated organizations, or those of the publisher, the editors and the reviewers. Any product that may be evaluated in this article, or claim that may be made by its manufacturer, is not guaranteed or endorsed by the publisher.

## Supplementary Material

Supplementary Figure 1 Immunoblots for all mice in the study.
