# Supplementary figures and images for: Helicobacter hepaticus Infection Promotes the Progression of Liver Preneoplasia in BALB/c Mice via the Activation and Accumulation of High-Mobility Group Box-1
Source: Front Microbiol. 2022 Jan 3;12:789752. doi: 10.3389/fmicb.2021.789752 (PMC8763329; doi:10.3389/fmicb.2021.789752)

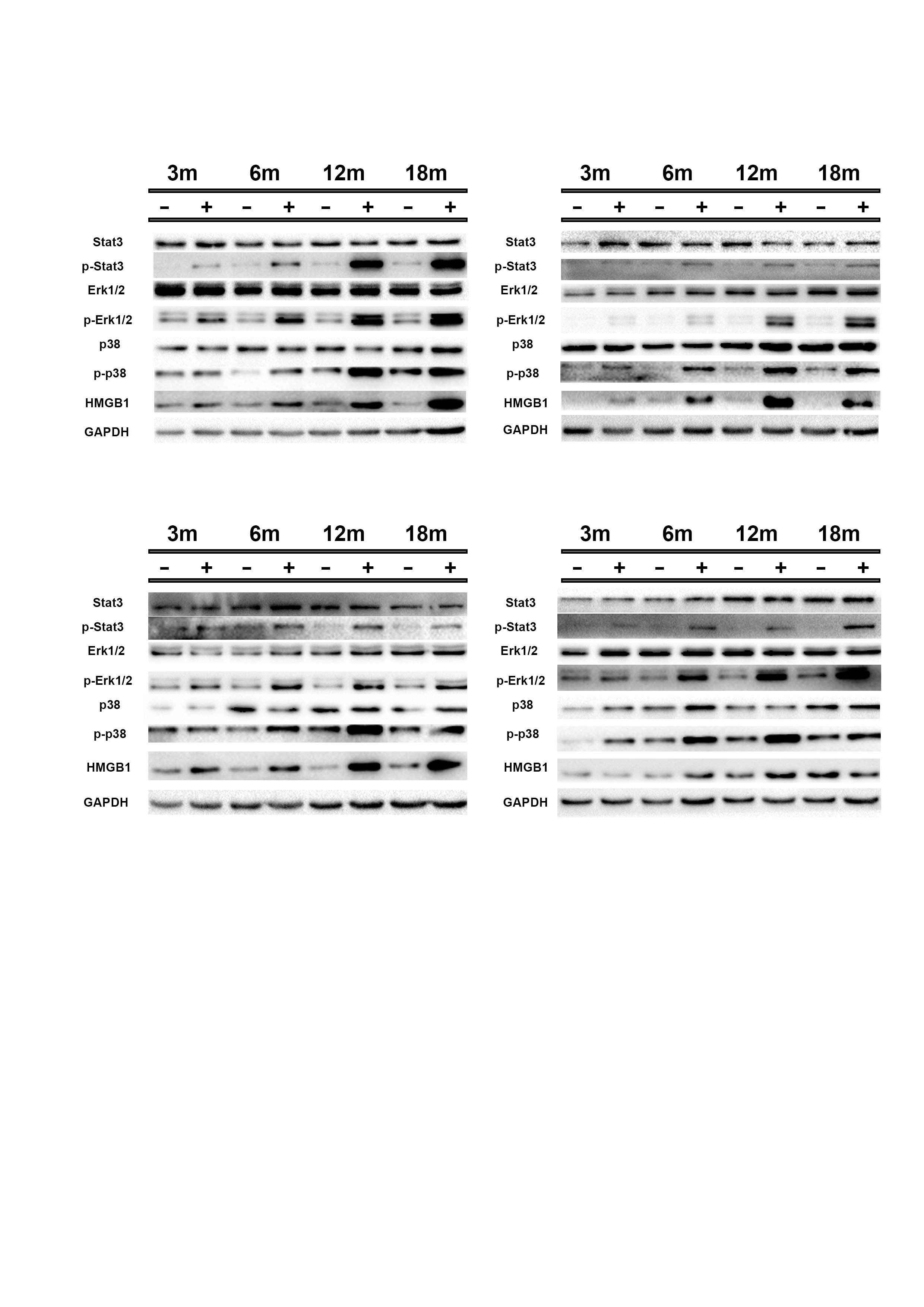

Supplement: Supplementary Figure 1 — Immunoblots for all mice in the study. [file Image_1.JPEG]
